# Bioinformatics and Computational Tools for Next-Generation Sequencing Analysis in Clinical Genetics

**DOI:** 10.3390/jcm9010132

**Published:** 2020-01-03

**Authors:** Rute Pereira, Jorge Oliveira, Mário Sousa

**Affiliations:** 1 Laboratory of Cell Biology, Department of Microscopy, Institute of Biomedical Sciences Abel Salazar (ICBAS), University of Porto (UP), 4050-313 Porto, Portugal; msousa@icbas.up.pt; 2 Biology and Genetics of Reproduction Unit, Multidisciplinary Unit for Biomedical Research (UMIB), ICBAS-UP, 4050-313 Porto, Portugal; jmoliveira@ibmc.up.pt; 3 UnIGENe and CGPP–Centre for Predictive and Preventive Genetics-Institute for Molecular and Cell Biology (IBMC), i3S-Institute for Research and Innovation in Health-UP, 4200-135 Porto, Portugal

**Keywords:** bioinformatics, clinical genetics, high throughput data, NGS pipeline, NGS platforms

## Abstract

Clinical genetics has an important role in the healthcare system to provide a definitive diagnosis for many rare syndromes. It also can have an influence over genetics prevention, disease prognosis and assisting the selection of the best options of care/treatment for patients. Next-generation sequencing (NGS) has transformed clinical genetics making possible to analyze hundreds of genes at an unprecedented speed and at a lower price when comparing to conventional Sanger sequencing. Despite the growing literature concerning NGS in a clinical setting, this review aims to fill the gap that exists among (bio)informaticians, molecular geneticists and clinicians, by presenting a general overview of the NGS technology and workflow. First, we will review the current NGS platforms, focusing on the two main platforms Illumina and Ion Torrent, and discussing the major strong points and weaknesses intrinsic to each platform. Next, the NGS analytical bioinformatic pipelines are dissected, giving some emphasis to the algorithms commonly used to generate process data and to analyze sequence variants. Finally, the main challenges around NGS bioinformatics are placed in perspective for future developments. Even with the huge achievements made in NGS technology and bioinformatics, further improvements in bioinformatic algorithms are still required to deal with complex and genetically heterogeneous disorders.

## 1. Introduction

Currently genetics is of extreme importance to medical practice as it provides a definitive diagnosis for many clinically heterogeneous diseases. Consequently, it enables a more accurate disease prognosis and provides guidance towards the selection of the best possible options of care for the affected patients. Much of its current potential derives from the capacity to interrogate the human genome at different levels from chromosomal to the single-base alterations.

**Figure 1:**
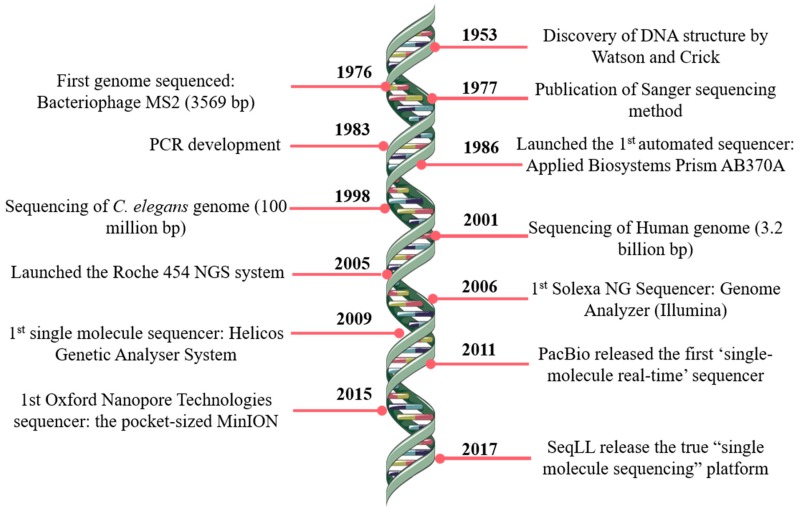
DNA sequencing timeline. Some of the most revolutionary and remarkable events in DNA sequencing. NG—next generation; PCR—polymerase chain reaction; SMS—single molecule sequencing; SeqLL—sequence the lower limit.

The pioneer works on DNA sequencing from Paul Berg [1], Frederick Sanger [2] and Walter Gilbert [3], made possible several progresses in the field, namely the development of a technique that opened totally new possibilities for DNA analysis, the Sanger’s ‘chain-termination’ sequencing technology, most widely known as Sanger sequencing [4]. Further technological developments marked the rising of DNA sequencing, allowing the launch of the first automated DNA sequencer (ABI PRISM AB370A) in 1986, which allowed the draft of the human genome during the following decade [5]. Since then, the progress continued and, essentially, over the last two decades decisive steps, namely in nanotechnology and informatics, contributed to the new generation of sequencing methods (Figure 1).

These new approaches targeted to complement and eventually replace Sanger sequencing. This technology is collectively referred to as next-generation sequencing (NGS) or massively parallel sequencing (MPS), which is often an umbrella to designate a wide diversity of approaches. Through this technology, it is possible to generate massive amounts of data per instrument run, in a faster and cost-effective way, streaming the parallel analysis of several genes or even the entire genome. Currently, the NGS market is expanding with the global market projected to reach 21.62 billion US dollars by 2025, growing about 20% from 2017 to 2025 (BCC Research, 2019). Thus, several brands are presently on the NGS market, with Illumina, Ion Torrent (Thermo Fischer Scientific), BGI Genomics, PacBio and Oxford Nanopore Technologies, being among the top sequencing companies. All provide different strategies towards the same problem, which is the massification of sequencing data. For simplicity, although not fully consensual classification among literature, one can consider that second-generation sequencing is based on massive parallel and clonal amplification of molecules (polymerase chain reaction (PCR)) [6]; whereas, the third-generation sequencing relies on single-molecule sequencing without a prior clonal amplification step [7,8,9].

In this review, a simplistic overview of the different steps involving the bioinformatic analysis of NGS data will be present and provide some insights concerning the main algorithms behind the analysis of this data.

## 2. NGS Library

In NGS, a library is defined as a collection of DNA/RNA fragments that represents either the entire genome/transcriptome or a target region. Each NGS platform has its specificities, but, in simple terms, the preparation of an NGS library starts with the fragmentation of the starting material, then sequence adaptors are connected to fragments to allow the enrichment of those fragments. A good library should have great sensitivity and specificity. This means that all fragments of interest should be equally represented in the library and should not contain random errors (non-specific products). However, it is easier said than done, as genomic regions are not equally prone to be sequenced, making the construction of a sensitive and specific library challenging [10].

The first step to prepare libraries in most NGS workflows is the fragmentation of nucleic acid. Fragmentation can be done either by physical or enzymatic methods [11,12]. Physical methods include acoustic shearing, sonication and hydrodynamic shear. The enzymatic methods include digestion by DNase I or Fragmentase. Knierim and co-works, compared both enzymatic and physical fragmentation methods and found similar yields, showing that the choice between physical or enzymatic method only relies on experimental design or external factors, such as lab facilities [13].

Once the starting DNA has been fragmented, adaptors are connected to those fragments. The adaptors are introduced to create known begins and ends to random sequences allowing the sequencing process. An alternative strategy was developed that combines fragmentation and adaptor ligation in a single step, thus making the process simpler, faster and requiring a reduce sample input. The process is known as tagmentation and is based on transposon-based technology [14].

Upon nucleic acid fragmentation, the fragments are select according to the desired library size. This is limited either by the type of NGS instrument and by the specific sequencing application. Short-read sequencers, such as Illumina and Ion Torrent, present best results when DNA libraries contain shorter fragments of similar sizes. Illumina fragments are longer than in Ion Torrent and can go up to 1500 bases in length [11] while in Ion Torrent the fragments can go up to 400 bases in length [15]. In contrast, long-read sequencers, like PacBio RS II [16] tend to produce ultra-long reads by fully sequencing a DNA fragment. The optimal library size is also limited by the sequencing application. For whole-genome sequencing, the longer fragments are preferable, while for RNA-seq and exome sequencing smaller fragments are feasible since most of the human exons are under 200 base pairs in length [17].

Next, an enrichment step is required, where the amount of target material is increased in a library to be sequenced. When just a part of the genome needs to be investigated both for research or clinical applications, it is known as target libraries. Basically, two methods are commonly used for such targeted approaches: capture hybridization-based sequencing and amplicon-based sequencing [18,19]. In the hybrid capture method, upon the fragmentation step, the fragmented molecules are hybridized specifically to DNA fragments complementary to the targeted regions of interest. This could be done by different methods such as microarray technology or using biotinylated oligonucleotide probes [20], which aims to physically capture and isolate the sequences of interest. Two well-known examples of commercial library prep solutions based on hybrid capture methods are the SureSelect (Agilent Technologies) and SeqCap (Roche).

Concerning the amplicon-based methods, those are based on the design of synthetic oligonucleotides (or probes), with a complementary sequence to the flanking regions of the target DNA to be sequenced. HaloPlex (Agilent Technologies) and AmpliSeq (Ion Torrent) are two examples of commercial library prep solutions based on amplicon-based strategies. The amplicon-based methods have the limitations intrinsic to PCR amplifications, such as bias, PCR duplicates, primer competition and non-uniform amplification of target regions (due to variation in GC content) [21]. Hybrid capture methods were shown to be superior to amplicon-based methods, providing much more uniform coverage and depth than amplicon assays [19]. However, hybridization methods have the drawback of higher costs due to the specificity of the method (cost of the probes, experimental design, software, etc.) and are more time consuming than amplicon approaches. Hence, several attempts have been performed to overcome PCR limitations. One promising strategy is the Unique molecular identifiers (UMIs) that are short DNA molecules, which are ligated to library fragments [22]. Those UMI have a random sequence composition that assures that every fragment with a UMI is unique in your library. This allows that after PCR enrichment, PCR duplicates can be found by searching for non-unique fragment-UMI combinations, while the real biological duplicated will contain those UMI sequences [23,24].

## 3. NGS Platforms

### 3.1. Second-Generation Sequencing Platforms

Second-generation platforms belong to the group of cyclic-array sequencing technologies (reviewed by [6]). The basic workflow for second-generation platforms includes the preparation and amplification of libraries (prepared from DNA/RNA samples), clonal expansion, sequencing, and analysis. The two most widely known sequencing companies of second-generation sequencing platforms are Illumina and Ion Torrent.

Illumina is a well-recognized American company that commercializes several integrated systems for the analysis of genetic variation and biological function that can be applied in multiple biological systems from agriculture to medicine. The process of Illumina sequencing is based on the sequencing-by-synthesis (SBS) concept, with the capture, on a solid surface, of individual molecules, followed by bridge PCR that allows its amplification into small clusters of identical molecules. Briefly, the DNA/cDNA is fragmented, and adapters are added to both ends of the fragments. Next, each fragment is attached to the surface of the flow cell by means of oligos on the surface that have a nucleotide sequence complementary to the adapters allowing the hybridization and the subsequent bridge amplification, forming a double-strand bridge. Next, it is denatured to give single-stranded templates, which are attached to the substrate. This process is continuously repeated and generates several million of dense clusters of double-stranded DNA in each channel of the flow cell. The sequencing can then occur, by the addition to the template (on the flow cell) of a single labeled complementary deoxynucleotide triphosphate (dNTP), which serves as a “reversible terminator”. The fluorescent dye is identified through laser excitation and imaging, and subsequently, it is enzymatically cleaved to allow the next round of incorporation. In Illumina, the raw data are the signal intensity measurements detected during each cycle [25]. Overall, this technology is considered highly accurate and robust but has some drawbacks. For instance, during the sequencing process, some dyes may lose activity and/or may occur a partial overlap between emission spectra of the fluorophores, which limit the base calling on the Illumina platform [26,27].

Ion Torrent, the major competitor of Illumina, employs a distinct sequence concept, named semi-conductor sequencing. The sequencing method in Ion Torrent is based on pH changes caused by the release of hydrogen ions (H^+^) during the polymerization of DNA. In Ion Torrent, instead of being attached to the surface of a flow cell, the fragmented molecules are bound to the surface of beads and amplified by emulsion PCR, generating beads with a clonally amplified target molecule (a molecule/bead). Each bead is then dispersed into a micro-well on the semiconductor sensor array chip, named as complementary metal-oxide-semiconductor (CMOS) chip [28,29]. Every time that the polymerase incorporates the complementary nucleotide into the growing chain, a proton is released, causing pH changes in the solution, which are detected by an ion sensor incorporated on the chip. Those pH changes are converted into voltage signals, which are then used to base calling. There are also limitations in this technology, with the major source of sequencing errors being the occurrence of homopolymeric template stretches. During the sequencing, multiple incorporations of the same base on each strand will occur, generating the release of a higher concentration of H^+^ in a single flow. The increased shift in pH generates a greater incorporation signal, indicating to the system that more than one nucleotide was incorporated. However, for longer homopolymers the system is not effective, making their quantification inaccurate [29,30].

### 3.2. Third-Generation Sequencers

The 3rd-generation NGS technology brought the possibility of circumventing common and transversal limitations of PCR-based methods, such as nucleotide misincorporation by a polymerase, chimera formation and allelic drop-outs (preferential amplification of one allele) causing an artificial homozygosity call. The first commercial 3rd-generation sequencer was the platform Helicos Genetic Analysis System [31,32]. In contrast to the second-generation sequencers, here the DNA was simply sheared, tailed with poly-A, and hybridized to a flow cell surface containing oligo-T nucleotides. The sequencing reaction occurs by the incorporation of the labeled nucleotide, which is captured by a camera. With this technology, every strand is uniquely and independently sequenced [31]. However, it was relatively slow and expensive and did not resist long in the market.

In 2011, the company Pacific Biosystems introduced the concept of single-molecule real-time (SMRT) sequencing, with the PacBio RS II sequencer [33]. Further, this technology enables the sequence of long reads (with average read lengths up to 30 kb) [33]. Individual DNA polymerases are attached to the zero-mode waveguide (ZMW) wells, which are nanoholes where a single molecule of the DNA polymerase enzyme can be directly placed [33]. A single DNA molecule is used as a template to polymerase incorporation of fluorescently labeled nucleotides. Each base has a different fluorescent dye, thereby emitting a signal out of the ZMW. A detector reads the fluorescent signal and, based on the color of the detected signal, identifies the signal. Then, the addition of the base leads to the cleavage of the fluorescent tag by the polymerase. A closed, circular single strand DNA (ssDNA) is used as template (called a SMRTbell) and can be sequenced multiple times to provide higher accuracy [33]. In addition, this technology enables *de novo* assembly and direct detection of haplotypes; high consensus accuracy and allows the epigenetic characterization (direct detection of DNA base modifications at one-base resolution) [7,34]. The first sequencers using the SMRT technology faced the drawbacks of having a limited high-throughput, higher costs and higher error rate. However, significant improvements have been achieved to overcome those limitations. More recently, PacBio launched the Sequel II System, which claims to reduce the project costs and timelines, comparing with the prior versions, with highly accurate individual long reads (HiFi reads, up to 175 kb) [35]. Using a PacBio System, Merker and co-workers claimed to be the first to demonstrate a successful application of long-read genome sequencing to identify a pathogenic variant in a patient with Mendelian disease, suggests that this technology has significant potential for the identification of disease-causing structural variation [36].

A second approach to single-molecule sequencing was commercialized by Oxford Nanopore Technologies (some authors refer to as fourth-generation) named MinION, commercially available in 2015 [37]. This sequencer does not rely on SBS but instead relies on the electrical changes in current as each nucleotide (A, C, T and G) passes through the nanopore [38]. Nanopore sequencing uses electrophoresis to transport an unknown sample through a small opening, then an ionic current pass through nanopores and the current is changed as the bases pass through the pore in different combinations. This information allows the identification of each molecule and to perform the sequencing [39]. In May 2017, Oxford Nanopore Technologies launched the GridION Mk1, a flexible benchtop sequencing and analysis device that offers real-time, long-read, high-fidelity DNA and RNA sequencing. It was designed to allow that up to five experiments run simultaneously or individually, with simple library preparation, enabling the generation of up to 150 Gb of data during a run [40]. This year, new advances were launched with PromethION 48 system that offers 48 flow cells, and each flow cell allows up to 3000 nanopores sequence in simultaneously, which can deliver up to 7.6 Tb yields in 72 h. Longer reads are of utmost importance to unveil repetitive elements and complex sequences, such as transposable elements, segmental duplications and telomeric/centromeric regions that are difficult to address with short reads [41]. This technology has already permitted the identification, annotation and characterization of tens of thousands of structural variants (SV) from a human genome [42]. Although the accuracy of nanopore sequencing is not yet comparable with that of short-read sequencing (for example, Illumina platforms claim 99.9% of accuracy), updates are constant and ongoing develops aim to expand the range of genomes and further improve accuracy of the technology [37,43].

The 10X Genomics was founded in 2012 and offers innovative solutions from single-cell analysis to complex SV and copy number variant analysis. In 2016, 10X Genomics launched the Chromium instrument that includes the gel beads in emulsion (GEMs) technology. In this technology, each gel bead is infused with millions of unique oligonucleotide sequences and mixed with a sample (that could be high molecular weight (HMW) DNA, individual cells or nuclei). Then, gel beads with the samples are added to an oil-surfactant solution to create gel beads in emulsion (GEMs). GEMs act as individual reaction vesicles in which the gel beads are dissolved and the sample is barcoded in order to create barcoded short-read sequences [44]. The advantage of GEMs technology is that it reduces the time, amount of starting material and costs [45,46]. For structural variant analysis, those short-read libraries are computationally reconstructed to be able to perform megabase-scale haplotype genome sequences using small amounts of input DNA. Zheng and co-workers have shown that this technology allows that linked-read phasing of SV distinguishes true SVs from false predictions and that has the potential to be applied to *de novo* genome assembly, remapping of difficult regions of the genome, detection of rare alleles and elucidation of complex structural rearrangements [45]. The chromium system also offers single-cell genome and transcriptional profiling, immune profiling and analysis of chromatin accessibility at single-cell resolution, with low error rates and high throughput [47,48]. Thus, opening exciting new applications especially on the development of new techniques for epigenetics research [49], *de novo* genome assembly [50] and for long sequencing reads [51].

## 4. NGS Bioinformatics

**Figure 2:**
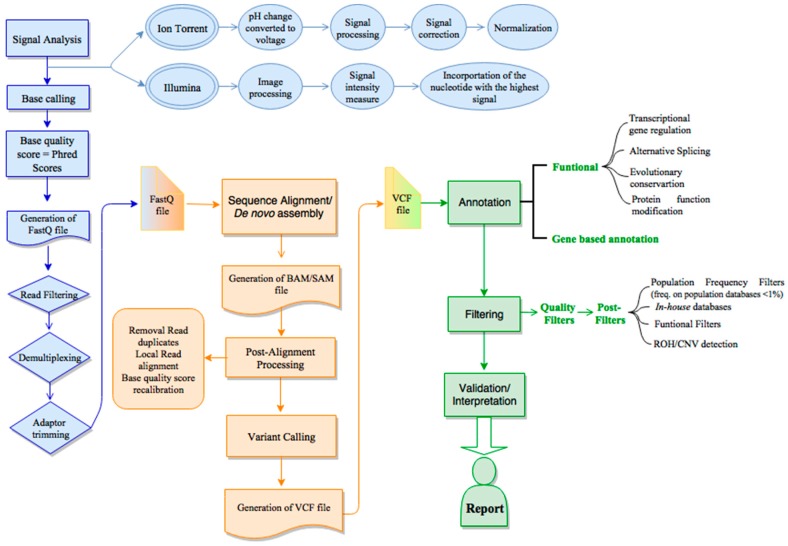
An overview of the next generation sequencing (NGS) bioinformatics workflow. The NGS bioinformatics is subdivided in the primary (blue), secondary (orange) and tertiary (green) analysis. The primary data analysis consists of the detection and analysis of raw data. Then, on the secondary analysis, the reads are aligned against the reference human genome (or *de novo* assembled) and the calling is performed. The last step is the tertiary analysis, which includes the variant annotation, variant filtering, prioritization, data visualization and reporting. CNV—copy number variation; ROH—runs of homozygosity, VCF—variant calling format.

As discussed above, the sequencing platforms are getting more efficient and productive, being now possible to completely sequence the human genome within one week at a relatively affordable price (PromethION promises the delivery of human genomes for less than $1000 [52]). Consequently, the amount of data generated demand computational and bioinformatics skills to manage, analyze and interpret the huge amount of NGS data. Therefore, a considerable development in NGS (bio)informatics is being performed, which could only take place greatly due the increasing computational capacities (hardware), as well as algorithms and applications (software) to assist all the required steps: from the raw data processing to more detailed data analysis and interpretation of variants in a clinical context. Typically, NGS bioinformatics is subdivided into the primary, secondary and tertiary analysis (Figure 2). The overall goal of each analysis is basically the same regardless of the NGS platform, however, each platform has its own particularities and specificities. For simplicity, we focused on the two main commercial 2nd-generation platforms: Illumina and Ion Torrent.

### 4.1. Primary Analysis

The primary data analysis consists of the detection and analysis of raw data (signal analysis), targeting the generation of legible sequencing reads (base calling) and scoring base quality. Typical outputs from this primary analysis are FASTQ file (Illumina) or unmapped binary alignment map (uBAM) file (Ion Torrent).

#### 4.1.1. Ion Torrent

**Figure 3:**
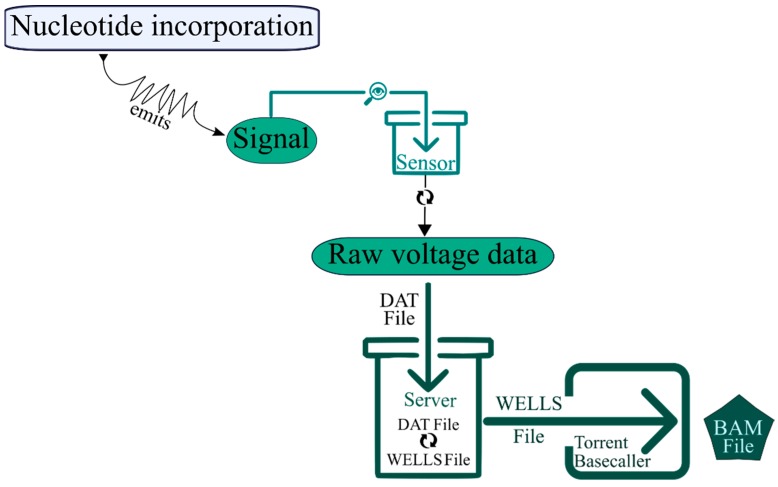
Schematic representation of the primary analysis workflow in Ion Torrent. Briefly, the signal emitted from nucleotide incorporation is inspected by the sensor, which converts the raw voltage data into a DAT file. This file serves as input to the server, which converts into a WELLS file. This last file is used as input on the Ion Torrent Basecaller module that gives a final BAM file, ready for the secondary analysis.

In the Ion Torrent platform, this task is basically performed in the Ion Torrent Suite Software [53]. As mentioned before, it starts with signal processing, in which the signal of nucleotide incorporation is detected by the sensor at the bottom of chip cell, converted to voltage and transferred from the sequencer to the server as a raw voltage data, named DAT file. For each nucleotide flow, one acquisition file is generated that contains the raw signal measurement in each chip well for the given nucleotide flow. During the analysis pipeline, these raw signal measurements are converted into incorporation measures, named WELLS file (Figure 3). The base calling is the final step of primary analysis and is performed by a base-caller module. Its objective is to determine the most likely base sequence, the best match for the incorporation signal stored in a WELLS file. The mathematical models behind this base-calling are complex (including heuristic and generative approaches) and comprising three sub-steps, namely: key sequence-based normalization, iterative/adaptative normalization and phase correction [53].

Such a procedure is required to address some of the errors occurring during the SBS process, namely phasing or signal droop (DR, which is signal decay over each flow, this is because some of the template clones attached to bead become terminated and there is no more nucleotide incorporation). Those errors occur quite frequently and thus, as an initial step, Ion Torrent performs phase-correction and signal decay normalization algorithms. The three parameters that are involved in the signal generation are the carry-forward (CF, that is, an incorrect nucleotide-binding), incomplete extension (IE, e.g., the flowed nucleotide did not attach to the correct position the template) and droop (DR). CF and IE regulate the rate of non-phase polymerase build-up, while DR measures DNA polymerase loss rate during a sequencing run. The chip area is divided into specific regions, and each well is further divided into two groups (odd- and even-numbered) each of which receives its own, independent set of estimates. Then, some training wells are selected and used to find optimal CF, IE and DR, using via the Nelder–Mead optimization algorithm [53]. This is a model that uses a triangle shape or a simplex, e.g., a generalized triangle in N dimensions, to search for an optimal solution in a multidimensional space [54].

The CF, IE and DR measurements, as well as, the normalized signals, are used by Solver, which follows the branch-and-bound algorithm. A branch-and-bound algorithm consists of a systematic listing of all partial sequences forming a rooted tree with the set of candidate solutions being placed as branches of this tree. The algorithm expands each branch by checking it against the optimal solution (the theoretical one) and goes on-and-on until finding the closer to the optimal solution [55]. Before the first performance of the solver, a key normalization needs to occur. The key normalization is based on the premise that the signal emitted during nucleotide incorporation is theoretically 1 and for the non-incorporation, the signal emitted is 0. Thus, the key normalization scales the measurements by a constant factor, which is selected to bring the signal of the known expected 1-mers produced by sequencing through the key as close to unity as possible. Once the solver has selected a base sequence, the predicted signal is used to create an adaptive normalization, which compares the theoretical with the real signal to subsequently generate an improved normalized signal with a reduced error rate [53]. This enters in a cycle of subsequent iterations (this means, applying a function repeatedly, with the output from one being the input of the next) with the solver for several rounds, allowing the normalized signal and the predicted signal to converge towards the best solution. This algorithm ends with a list of promising partial sequences, from which estimates to determine the most likely base sequence in the well.

#### 4.1.2. Illumina

**Figure 4:**
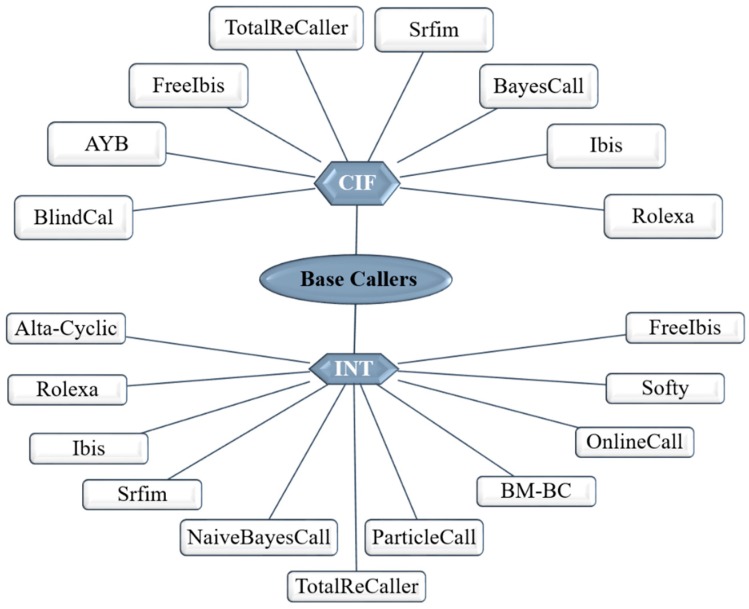
Summary of some widely used base callers’ software available for the Illumina platform. The software is grouped according to the input file: INT (intermediate executable code) text format for the older tools and CIF (cluster intensity files) for the most recent platforms.

As for the Illumina platform, the principle for signal detection relies on fluorescence. Therefore, the base-calling is apparently much simpler, is made directly from fluorescent signal intensity measurements resulting from the incorporated nucleotides during each cycle. Illumina claims that their SBS technology delivers the highest percentage of error-free reads. The latest versions of their chemistry have been reoptimized to enable accurate base calling in difficult genomic regions such as GC rich, homopolymers and of repetitive nature [56]. Further, the dNTPs have been chemically modified to contain a reversible blocking group that acts as a temporary terminator for DNA polymerization. After each dNTP incorporation, the image is processed to identify the corresponding base and then enzymatically cleaved-off to allow incorporation of the next one [56]. However, a single flow cell often contains billions of DNA clusters tightly and randomly packed into a very small area. Such physical proximity could lead to crosstalk events between neighboring DNA clusters. As fluorophores attached to each base produce light emissions, there can be some degree of interference between the nucleotide signals, which can overlap with the optimal emissions of the fluorophores of the surrounding clusters. Thus, although the base calling is simpler than in Ion Torrent, the image processing step is quite complex. The overall process requires aligning each image to the template of cluster position on the flow cell, image extraction to assign an intensity value for each DNA cluster, followed by intensity correction. Besides this crosstalk correction, another problematic aspect occurs during the sequencing process and influence the base-calling process such as phasing (failures in nucleotide incorporation), fading (or signal decay) and T accumulation (thymine fluorophores are not always efficiently removed after each iteration, causing a build-up of the signal along the sequencing run). Over many cycles, these errors will accumulate and decrease the overall signal to noise ratio per single cluster, causing a decrease in quality towards the ends of the reads [27]. Some of the initial base-callers for the Illumina platform were Alta-Cyclic [57] and Bustard [58]. Currently, there are multiple other base-callers differing in the statistical and computational methodologies used to infer the correct base [59,60,61]. Despite this variability (Figure 4), the most widely used base-caller is the Bustard and several base-calling algorithms were built using Bustard as the starting point. Overall, the Bustard algorithm is based on fluorescence signals conversion into actual sequence data. The intensities of four channels for every cluster in each cycle are taken, which allows the determination concentration of each base. The Bustard algorithm is based on a parametric model and applies the Markov algorithm to determine transition matrix modeling probability of phasing (no new base synthesized), prephasing (two new bases synthesized) and normal incorporation. The Bustard algorithm assumes a crosstalk matrix constant for a given sequencing run and that phasing affects all nucleotides in the same way [58]. Aiming to improve performance and decreasing error rate, several base-callers have been developed, however, there is no evidence that a given base caller is better than the other [59,60]. Comparison between the performance of different base-callers, namely regarding the alignment rates, error rate and running time, shows that AYB presents the lowest error rate, the BlindCall is the fastest while BayesCal has the best alignment rate. BayesCall, freeIbis, Ibis, naiveBayesCall, Softy FB and Softy SOVA did not show significative differences among each other, but all showed improvements in the error rates comparing to the standard Bustard [60]. Recently, was developed the base-caller 3Dec for Illumina sequencing platforms, which claims to reduce the base-calling errors by 44–69% comparing to the previous ones [61].

#### 4.1.3. Quality Control: Read Filtering and Trimming

As errors occur both in Ion Torrent and Illumina sequencing, these are expressed in quality scores of the base call based using Phred score, a logarithmic error probability. Thus, a Phred score of 10 (Q10) refers to a base with a 1 in 10 probability of being incorrect or an accuracy of 90.0%, and as for Q30 means 1 in 1000 probability of an incorrect base or 99.9% accuracy [62]. Fastq files are important to the first quality control step, as contains all the raw sequencing reads, the filenames and the quality values, with higher numbers indicative of higher qualities [63]. The quality of the raw sequence is critical for the overall success of NGS analysis, thereby several bioinformatic tools were developed to evaluate the quality of raw data, such as the NGS QC toolkit [64], QC-Chain [65] and FastQC [66]. FastQC is one of the most popular. As output, FastQC gives a report containing well-structured and graphically illustrated information about different aspects of the read quality. If sequence reads have enough quality the sequences are ready to be aligned/mapped against the reference genome. The Phred score is also useful to filter and trimming sequences. Additional trimming is performed at the ends of each read to remove adapters’ sequences. Overall the trimming step, although reducing the overall number and the length of reads, it raises quality to acceptable levels. Several tools were developed to perform trimming, namely with Illumina data, such as BTrim [67], IeeHom [68], AdapterRemoval [69] and Trimmonatic [70]. The choice of the tool is highly dependent on the dataset, downstream analysis and parameters used [71]. In Ion Torrent, sequencing and data management are processed in Torrent Suite Software, which has a Torrent Browser as a web interface. To further analyze the sequences, a demultiplexing process is required, which separates the sequencing reads into separate files according to the barcodes used for each sample [72]. Most of the demultiplexing protocols are specific to NGS platform manufacturers.

Demultiplexing is the followed adapter trimming step, whose function is the removal of the remaining library adapter sequences from the end of the reads, in most cases from 3’end, but can depend on the library preparation. This step is necessary, since residual adaptor sequences in the reads may interfere with mapping and assembly. Fabbro and co-works showed that trimming is important namely in RNA-Seq, SNP identification and genome assembly procedures, increasing the quality and reliability of the analysis, with further gains in terms of execution time and computational resources needed [71]. Several tools are used to perform the trimming, namely with Illumina data. Again, there is no best tool, instead, the choice is dependent on downstream analysis and user-decided parameter-dependent trade-offs [71]. In Ion Torrent, this is also done in Torrent Suite^TM^ Software as well.

### 4.2. Secondary Analysis

The next step of the NGS data analysis pipeline is a secondary analysis, which includes the reads alignment against the reference human genome (typically hg19 or hg38) and variants calling. To map sequencing reads two different alternatives can be followed: read alignment, that is the alignment of the sequenced fragments against a reference genome, or *de novo* assembly that involves assembling a genome from scratch without the help of external data. The choice between one approach or others could simply rely on the existence or not of a reference genome [73]. Nevertheless, for most NGS applications, namely in clinical genetics, mapping against a reference sequence is the first choice. As for *de novo* assembly, it is still mostly confined to more specific projects, especially targeting to correct inaccuracies in the reference genome [74] and to improve the identification of SV and other complex rearrangements [36].

Sequence alignment is a classic problem addressed by bioinformatics. Sequencing reads from most NGS platforms are short, therefore to sequence a genome, billions of DNA/RNA fragments are generated that must be assembled, like a puzzle. This represents a great computational challenge, especially when dealing with the existence of reads derived from repetitive elements, which at an extreme condition the algorithm must choose from which repeat copy the read belongs to. In such a context it is impossible to make high-confidence calls, the decision must be taken either by the user or software through a heuristic approach. Sequence alignment errors may emerge from multiple reasons. To start, errors in sequencing (caused by a process such as fading and signal droop, as previously discussed), as well as, discrepancies between the sequenced data and the reference genome also cause misalignment problems. Another major difficulty is to establish a threshold between what is a real variation and a misalignment. The most widely accepted data input file format for an assembly is FASTQ [66]. As output, the typical files are the binary alignment/map (BAM) and sequence alignment/map (SAM) format in from various sequencing platforms and read aligners. Both include basically the same information, namely read sequence, base quality scores, location of alignments, differences relative to reference sequence and mapping quality scores (MAPQ). The main distinction between them is that SAM format is a text file, created to be informatically easier to process with simple tools, while BAM format provides binary versions of the same data [75]. Alignments can be viewed using user-friendly and freely available software, such as the Interactive Genome Viewer (IGV) [76] or Genome Browse (http://goldenhelix.com/products/GenomeBrowse/index.html).

#### 4.2.1. Sequence Alignment

The preferential assemble method when the reference genome is known is the alignment against the reference genome. A mapping algorithm will try to locate a location in the reference sequence that matches the read, tolerating a certain number of mismatches to allow subsequence variation detection. More than 60 tools for genome mapping have been developed and as the NGS platforms are updated more and more tools will appear being an on-going evolving process (for further detail see [77,78]). Among the commonly used methods to perform short reads alignments, we highlighted Burrows–Wheeler Aligners (BWAs) and Bowtie mostly used for Illumina; whereas for Ion Torrent, the Torrent Mapping Alignment Program (TMAP) is the recommended alignment software as it was specifically optimized for this platform.

BWA uses the Burrows–Wheeler transform algorithm (a data transformation algorithm that restructures data to be more compressible), initially developed to prepare data for compression techniques such as bzip2, it is a fast and efficient aligner performing very well for both short and long reads [79]. Bowtie (now Bowtie 2) has the advantage of being faster than BWA for some types of alignment [80], but it may compromise the quality, namely reduction of sensitivity and accuracy. Bowtie may fail to align some reads with valid mappings when configured for maximum speed. It is usually applied to align reads derived from RNA sequencing experiments. Ruffalo and co-workers, developed a simulation and evaluation suite, Seal, to simulate runs in order to compare the most widely used tools for mapping, such as Bowtie, BWA, mr- and mrsFAST, Novoalign, SHRiMP and SOAPv2 [81]. The study compared different parameters, including sequencing error, indels and coverage and concluded that there is no perfect tool. Each presents different specificities and performances that are dependent on the users’ choice, e.g., what has priority the accuracy of the coverage, despite all the analyzed tools being independent of a specific platform [81]. The specific ion torrent tool, TMAP comprises two stages: (i) initial mapping (using Smith–Waterman or Needleman–Wunsch algorithms) allowing reads to be roughly aligned against the reference genome and (ii) alignment refinement, as particular positions of the read are realigned to the corresponding position in the reference. This alignment refinement is designed to compensate for specific systematic biases of the Ion Torrent sequencing process (such as homopolymer alignment and phasing errors with low indel scores) [82].

##### *De novo* Assembly

*De novo* assembly circumvent the bias from a reference genome, limitations of inaccuracies in the reference genome, being the most effective to identify SV and complex rearrangements, avoiding the loss of new sequences. Most of the algorithms for *de novo* assembly are based on an overlap-layout strategy, in which equal regions in the genome are identified and then overlapped by fragmented sequenced ends. This strategy has the limitation of being prone to incorrect alignment, especially with short read lengths, mainly due to the highly repetitive regions making difficult to identify which region of the genome they belong to. *De novo* assembly is preferred when longer reads are available. Further *de novo* assembly is much slower and requires more computational resources (such as memory) comparing to mapping assemblies [73].

As mentioned *de novo* assemblers are mostly based on graphs theory and can be categorized into three major groups (Figure S1): (i) the Overlap-Layout-Consensus (OLC), (ii) the de Bruijn graph (DBG, also known as Eurelian) methods using some form of K-mer graph and (iii) the greedy graph algorithms that can use either OLC or DBG [78].

Briefly, the greedy algorithm starts by adding a read to another identical one. This process is repeated until all options of assembly for that fragment is achieved and is repeated to all fragments. Each operation uses the next highest-scoring overlap to make the next join. To make the scoring the algorithm measures, for instance, the number of matching bases in the overlap. This algorithm is suitable for a small number of reads and smaller genomes. In contrast, the OLC method was optimized for the low-coverage long reads [78,83]. The OLC begins by identifying the overlaps between pairs of reads and builds a graph of the relationships between those reads, which is highly computationally demanding, especially with a high number of reads. As soon as a graph is constructed, a Hamiltonian path (a path in an undirected/directed graph that visits each vertex exactly once) is required, which give rise to contig sequences. To end the process a further computational and manual inspection is required [83,84]. The Eurelian (or DBG method) assembler is particularly suitable for representing the short-read overlap relationship. Here a graph is also constructed, however, the nodes of the graph represent k-mers and the edges represent adjacent k-mers that overlap by k-1 bases. Hence, the graph size is determined by the genome size and repeat content of the sequenced sample, and in principle, will not be affected by the high redundancy of deep read coverage [85].

With long-range sequencing and mapping technologies, the third-generation sequencers, newer bioinformatics tools are continuously being created that leverage the unique features and overcome the limitations of these new sequencing and mapping platforms, such as high error rates dominated by false insertions or deletions and sparse sequencing rather than true long reads. Some of those methods were deeply reviewed elsewhere [86].

#### 4.2.2. Post-Alignment Processing

**Figure 5:**
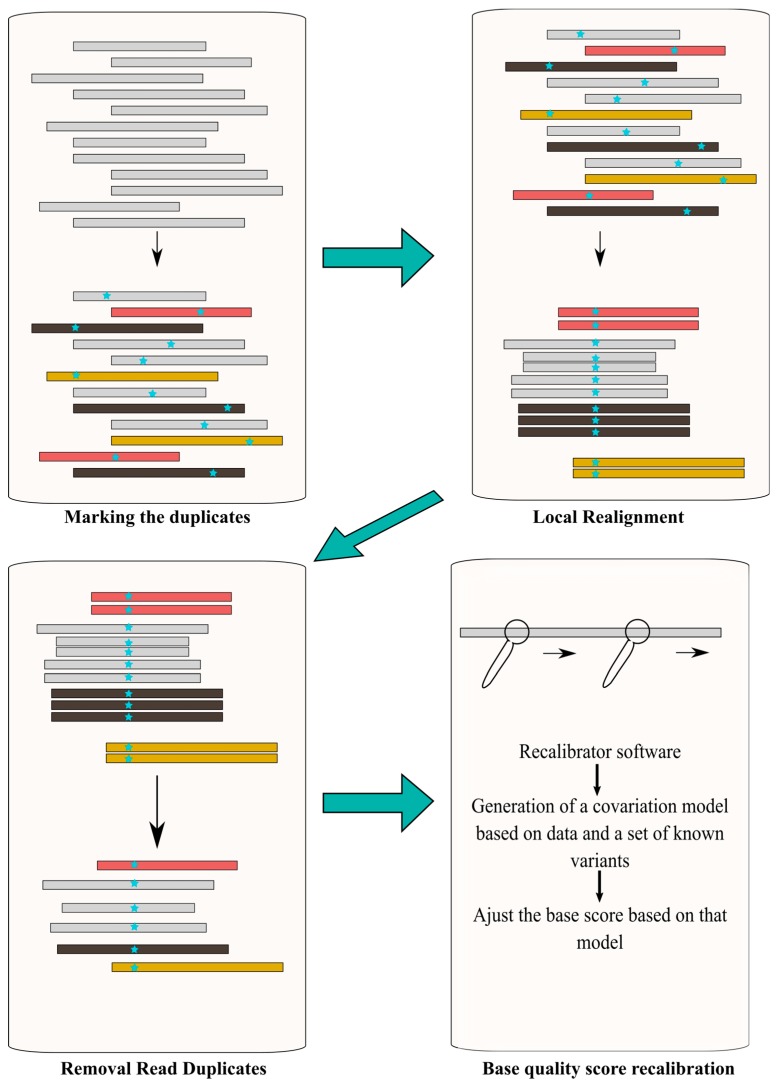
Schematic representation of the main steps involved in the post-alignment process.

Post-alignment processing is recommended prior to performing the variant call. Its objective is to increase the variant call accuracy and quality of the downstream process, by reducing base call and alignment artifacts [87]. In the Ion Torrent platform, this is included in TMAP software, whereas in Illumina other tools are required. In general terms, it consists of filtering (removal) of duplicates reads, intensive local realignment (mostly near INDELs) and base quality score recalibration [88] (Figure 5).

SAMtools [75], Genome Analysis Toolkit (GATK) [89] and Picard (http://broadinstitute.github.io/picard/) are some of the bioinformatic tools used to perform this post-alignment processing. Since variant calling algorithms assume that, in the case of fragmentation-based libraries, all reads are independent, removal of PCR duplicates and non-unique alignments (i.e., reads with more than one optimal alignment) is critical. This step can be performed using Picard tools (e.g., MarkDuplicates). If not removed, a given fragment will be considered as a different read, increasing the number of incorrect variant calls and leading to an incorrect coverage and/or genotype assessment [88]. Reads spanning INDELs impose further processing. Given the fact that each read is independently aligned to the reference genome when an INDEL is part of the read, there is a higher change for alignment mismatches. The realigner tool firstly determines suspicious intervals requiring a realignment due to the presence of INDELs, next the realigner runs over those intervals combining shreds of evidence to generate a consensus score to support the presence of the INDEL [88]. IndelRealigner from the GATK suite can be used to run this step. As mentioned, the confidence of the base call is given by the Phred-scaled quality score, which is generated by the sequencer machine and represents the raw quality score. However, this score may be influenced by multiple factors namely the sequencing platform and the sequence composition, and not reflecting the base-calling error rate [90]. Consequently, is necessary to recalibrate this score to improve variant calling accuracy. BaseRecalibrator from the GATK suite is one of the most commonly used tools.

#### 4.2.3. Variant Calling

The variant call step has the main objective of identifying variants using the post-processed BAM file. Several tools are available for variant calling, some identify variants based on the number of high confidence base calls that disagree with the reference genome position of interest. Other, use Bayesian, likelihood, or machine learning statistical methods that use factor parameters, such as base and mapping quality scores, to identify variant differences. Machine learning algorithms have evolved greatly in recent years and will be critical to assist scientists and clinicians to handle large amounts of data and to solve complex biological challenges [91,92].

SAMtools, GATK and Freebayes belong to the latter group and are among the most widely used toolkits for Illumina data [93]. Ion Torrent has its own variant caller known as the Torrent Variant Caller (TVC). Running as a plugin in the Ion Torrent server, TVC calls single-nucleotide polymorphisms (SNVs), multi-nucleotide variants (MNVs), INDELS in a sample across a reference or within a targeted subset of that reference. Several parameters can be customized, but often predefined configurations (germ-line vs. somatic, high vs. low stringency) can be used depending on the type of experiment performed.

Most of these tools use the SAM/BAM format as input and generate a variant calling format (VCF) file as their output [94]. The VCF format is a standard format file, currently in version 4.2, developed by the large sequencing projects such as the 1000 genomes project. VCF is basically a text file contains meta-information lines, a header line, followed by data lines each containing information chromosomal position, the reference base, the identified alternative base or bases. The format also contains genotype information on samples for each position [95]. VCFtools provide the possibility to easily manipulate VCF files, e.g., merge multiple files or extracting SNPs from specific regions [94].

##### Structural Variants Calling

**Figure 6:**
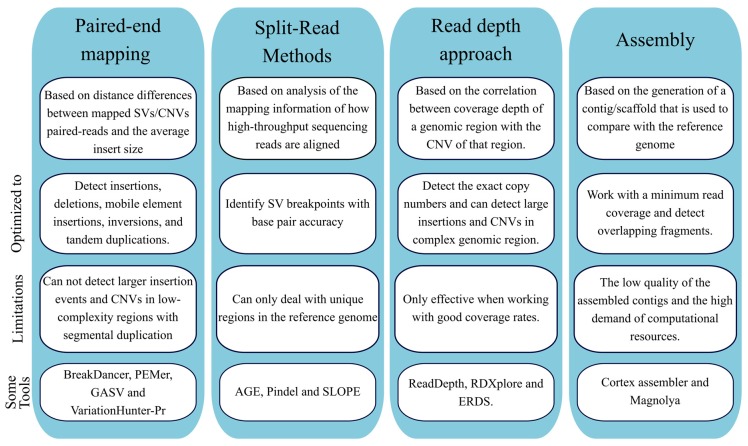
Summary of the main methods for calling structural variants (SV) and copy number variation (CNV) from next generation sequencing (NGS) data.

Genetic variations can occur in the human genome ranging from SNV and INDELS to more complex (submicroscopic) SV [96]. These SV include both large insertions/duplications and deletions (also known as copy number variants, CNVs) and large inversions and can have a great impact on health [97]. Longer-read sequencers hold the promise to identify large structural variations and the causative mutations in unsolved genetic diseases [36,98,99]. Incorporating the calling of such SV would increase the diagnostic yield of these NGS approaches, overcoming some of the limitations present in other methods and with the potential to eventually replace them. Reflecting this growing tendency, several bioinformatic tools have been developed to detect CNVs from NGS data. At the moment to detect CNVs from NGS data five approaches can be used, according to type algorithms/strategies used (Figure 6): paired-end mapping, split read, read depth, *de novo* genome assembly and combinatorial approach [100,101].

The paired-end mapping algorithm compares the average insert size between the present sequenced read-pairs with the expected size based on the reference genome. Discordant reads maps may indicate either the presence of deletion or insertion. The paired-end mapping methods can also efficiently identify mobile element insertions, insertions (smaller than the average insert size of the genome library inversions), and tandem duplications [100]. A limitation of paired-end mapping is the inability to detect CNVs in low-complexity regions with segmental duplications [102]. BreakDancer [103] and PEMer [102] are two examples of tools using the paired-end mapping strategy. As for split read methods, derives from the concept that due to a structural variant only one of the reads of the pair is correctly aligned to the reference genome, while the other one fails to map or only partially aligns [100]. The latter reads also have the potential to provide the accurate breakpoints of the underlying genetic defect at the base-pair level. The partially mapped reads are spliced into multiple fragments that are aligned to the reference genome independently [100,101]. This further remapping step provides the exact starting and ending positions of the insertion/deletion events. Some example of tools using the split read method is Pindel [104] and SLOPE [105]. As for the read depth methodology, it starts from the assumption that is possible to establish a correlation between the number of copies and the read coverage depth. The study design, as for normalization or data comparison, can be based either in single samples, paired cases, control samples or a large dataset (population) of samples. In general terms, these algorithms present for main steps: mapping, normalization, estimation of copy number and segmentation [100]. First, reads are aligned, and coverage is estimated across an individual genome in predefined intervals. Next, the CNV-calling algorithm must perform a normalization in terms of the number of reads to compensate potential biases, for instance, due to GC content or repetitive sequences. Normalization of CNV results is challenging due to natural CNV variations. This problem is even more aggravated in cancer, as cancers have a huge spectrum of CNV tumor types within, as well as the cell-to-cell variations [106,107]. Having a normalized read depth, it is possible to obtain a rough estimation of copy numbers (gains or losses) across genomic segments. As for the final step, segmentation, regions with similar copy number are merged to detect abnormal copy number [100,108]. In comparison with pair-end and split reads methods, read depth approaches have the advantage to estimate the number copies of CNVs and a tendency to obtain better performances with larger CNVs [100].

Detection of CNV mainly relies on whole-genome sequencing (WGS) data since includes non-coding regions which are known to encompass a significant percentage of SV [109]. Whole-exome sequencing (WES) has emerged as a more cost-effective alternative to WGS and the interest in detecting CNV from WES data has grown considerably. However, since only a small fraction of the human genome is sequenced by WES it is not able to detect the complete spectrum of CNVs. The lower uniformity of WES as compared with WGS may reduce its sensitivity to detect CNVs. Usually, WES generates higher depth for targeted regions as compared with WGS. Consequently, most of the tools developed for CNVs detection using WES data, have depth-based calling algorithms implemented and require multiple samples or matched case-control samples as input [100]. Ion Torrent also developed a proprietary algorithm, as part of the Ion Reporter software, for the detection of CNVs in NGS data derived from amplicon-based libraries [110].

### 4.3. Tertiary Analysis

The third main step of the NGS analysis pipeline addresses the important issue of “making sense” or data interpretation, that is finding, in the human clinical genetics context, the fundamental link between variant data and the phenotype observed in a patient. The tertiary analysis starts with variant annotation, which adds a further layer of information to predict the functional impact of all variants previously founded in variant calling steps. Variant annotation is followed by variant filtering, prioritization and data visualization tools. These analytical steps can be performed by a combination of a wide variety of software, which should be in a constant update to include the recent scientific findings, requiring constant support and further improvements by the developers.

#### 4.3.1. Variant Annotation

The variant annotation is a key initial step for the analysis of sequencing variants. As mentioned before, the output of the variant calling is a VCF file. Each line in such a file contains high-level information about a variant, such as genomic position, reference and alternate bases, but no data about its biological consequences. Variant annotation offers such biological context for the all variants found. Given the massive amount of NGS data, data annotation is performed automatically. Several tools are currently available, and each uses different methodologies and databases for variant annotation. Most of the tools can perform both the annotation of SNVs and the annotation of INDELs, whereas annotation SV or CNVs are more complex and are not performed by all methods [111]. One basic step in the annotation is to provide the variant’s context. That is in which gene the variant is located, its position within the gene and the impact of the variation (missense, nonsense, synonymous, stop-loss, etc.). Such annotation tools offer additional annotation based on functionality, integrating other algorithms such as SIFT [112], PolyPhen-2 [113], CADD [114] and Condel [115], which computes the consequence scores for each variant based on various different parameters, like the degree of conservation of amino acid residues, sequence homology, evolutionary conservation, protein structure or statistical prediction based on known mutations. Additional annotation can resource to disease variants databases such as ClinVar and HGMD, where information about its clinical association is retrieved. Among the extensive list of annotation tools, the most widely used are ANNOVAR [116], variant effect predictor (VEP) [117], snpEff [118] and SeattleSeq [119]. ANNOVAR is a command-line tool that can identify SNPs, INDELs and CNVs. It annotates the functional effects of variants with respect to genes and other genomic elements and compares variants to existing variation databases. ANNOVAR can also evaluate and filter-out subsets of variants that are not reported in public databases, which is important especially when dealing with rare variants causing Mendelian diseases [120]. Like ANNOVAR, VEP from Ensembl (EMBL-EBI) can provide genomic annotation for numerous species. However, in contrast with ANNOVAR, that requires software installation and experienced users, VEP has a user-friendly interface through a dedicated web-based genome browser, although it can have programmatic access via a standalone Perl script or a REST API. A wider range of input file formats are supported, and it can annotate SNPs, indels, CNVs or SVs. VEP searches the Ensembl Core database and determines where in the genomic structure the variant falls and depending on that gives a consequence prediction. SnpEff is another widely used annotation tool, standalone or integrated with other tools commonly used in sequencing data analysis pipelines such as Galaxy, GATK and GKNO projects support. In contrast with VEP and ANNOVAR, it does not annotate CNVs but has the capability to annotate non-coding regions. It can perform annotation for multiple variants being faster than VEP [118].

The variant annotation may seem like a simple and straightforward process; however, it can be very complex considering the genetic organization’s intricacy. In theory, the exonic regions of the genome are transcribed into RNA, which in turn is translated into a protein. Making that one gene would originate only one transcript and ultimately a single protein. However, such a concept (one gene–one enzyme hypothesis) is completely outdated as the genetic organization and its machinery are much more complex. Due to a process is known as alternative splicing, from the same gene, several transcripts and thus different in proteins can be produced. Alternative splicing is the major mechanism for the enrichment of transcriptome and proteome diversity [121]. While imperative to explain genetic diversity, when considering annotation this is a major setback, depending on the transcript choice the biological information and implications concerning the variant can be very different. Additional blurriness concerning annotation tools is caused by the existence of a diversity of databases and reference genomes datasets, which are not completely consistent and overlapping in terms of content. The most frequently used are Ensembl (http://www.ensembl.org), RefSeq (http://www.ncbi.nlm.nih.gov/RefSeq/) and UCSC genome browser (http://genome.ucsc.edu), which contain such reference datasets and additional genetic information for several species. These databases also contain a compilation of the different sets of transcripts that were observed for each gene and are used for variant annotation. Each database has its own particularities and thus depending on the database used for annotation the outcome may turn different. For instance, if for a given locus one of the possible transcripts has an intron retained, while in the others have not, a variant located in such region will be considered as located in the coding sequencing in only one of the isoforms. In order to minimize the problem of multiple transcripts, the collaborative consensus coding sequence (CCDS) project was developed. This project aims to catalog identical protein annotations both on human and mouse reference genomes with stable identifiers and to uniformize its representation on the different databases [122]. The use of different annotations tools also introduces more variability to NGS data. For instance, ANNOVAR by default uses 1 Kb window to define upstream and downstream regions [120], while SnpEff and VEP use 5 kb [117,118]. This makes the classification of variant different even though the same transcript was used. McCarthy and co-workers found significant differences in VEP and ANNOVAR annotations of the same transcript [123]. Besides the problems related to multiple transcripts and annotation tools, there are also problems with overlapping genes, i.e., more than one gene in the same genomic position. There is still no complete/definitive solution to deal with these limitations, thus results from variant annotation should be analyzed with respect to the research context problem and, if possible, resorting to multiple sources.

#### 4.3.2. Variant Filtering, Prioritization and Visualization

After annotation of a VCF file from WES, the total number of variants may range between 30,000 and 50,000. To make clinical sense of so many variants and to identify the disease-causing variant(s), some filtering strategies are required. Although quality control was performed in previous steps, several false-positive variants are still present. Consequently, when starting the third-level of NGS analysis, it is highly recommended to, based on quality parameters or previous knowledge of artifacts, reduce the number of false-positive calls and variant call errors. Parameters such as the total number of independent reads and the percentage of reads showing the variant, as well as, the homopolymer length (particularly for Ion Torrent, with stretches longer than five bases being suspicious) are examples of filters that could be applied. The user should define the threshold based on observed data and research question but, relatively to the first parameter, less than 10 independent reads are usually rejected since it is likely due to sequencing bias or low coverage.

One of the more commonly used NGS filters is the population frequency filter. Minor allele frequency (MAF), one of the metrics used to filter based on allele frequency, can sort variants in three groups: rare variants (MAF < 0.5, usually selected when studying Mendelian diseases), low frequent variants (MAF between 0.5% and 5%) and common variants (MAF > 5%) [124]. Populational databases that include data from thousands of individuals from several populations represent a powerful source of variant information about the global patterns of human genetic variation. It also helps, not only to better identify disease alleles but also are important to understand the populational origins, migrations, relationships, admixtures and changes in population size, which could be useful to understand some diseases pattern [125]. Population databases such 1000 genome project [126], Exome Aggregation Consortium (ExAC) [127], and the Genome Aggregation Database (gnomAD; http://gnomad.broadinstitute.org/) are the most widely used databases. Nonetheless, this filter has also limitations and could cause erroneous exclusion, which is difficult to overcome. For instance, as carriers of recessive disorders carriers do not show any signs of the disease, the frequency of damaging alleles in populational variant databases can be higher than the established threshold. In-house variant databases are important to assist variant filtering, namely, to help to understand the frequency of variants in a study population and to identify systematic errors of an in-house system.

Numerously standardized widely accepted guidelines for the evaluation of genomic variations obtained through NGS such as the American College of Medical Genetics and Genomics (ACMG) and the European Society of Human Genetics guidelines. These provide standards and guidelines for the interpretation of genomic variations [128,129].

In kindred with a recognizable inheritance pattern, it is advisable to perform family inheritance-based model filtering. These are especially useful if more than one patient of such families is available for study, as it would greatly reduce the number of variants to be thoroughly analyzed. For instance, for diseases with an autosomal dominant (AD) inheritance pattern the ideal situation would be testing at least three patients, each from a different generation, and select only the heterozygous variants located in the autosomes. If a pedigree indicates a likely x-linked disease, variants located in the X chromosome are select and those in other chromosomes are not primarily inspected. As for autosomal recessive (AR) diseases, with more than one affected sibling, it would be important to study as many patients as possible and to select homozygous variants in patients that were found in heterozygosity in both parents, or genes with two heterozygous variants with distinct parental origins. For sporadic cases (and cases in which the disease pattern is not known), the trio analysis can constitute as extremely useful to reduce the analytical burden. In such a context, heterozygous variants found only in the patient and not present in both parents would indicate a *de novo* origin. Even in non-related cases with very homogeneous phenotypes, such as those typically syndromic, it is possible to use an overlap-based strategy [130], assuming that the same gene or even the same variant is shared among all the patients. An additional filter, useful when many variants persist after applying others, is based on the predicted impact of variants (functional filter). In some pipelines intronic or synonymous variants, based on the assumption that they likely to be benign (non-disease associated). Nonetheless, care should be taken since numerous intronic and apparent synonymous variants, have been implicated in human diseases [131,132]. Thus, a functional filter is applied in which the variants are prioritized based on its genomic location (exonic or splice-sites). Additional information for filtering missense variants includes evolutionary conservation, predicted effect on protein structure, function or interactions. To enable such filtering the scores generated by algorithms to evaluate missense variants (for instance PolyPhen-2, SIFT and CADD) are annotated in the VCF. The same applies to variants that might have an effect over splicing, as prediction algorithms are being incorporated in VCF annotation, such as the Human Splice finder [133] in VarAFT [134] (some more examples in Table 1).

Although functional annotation adds an important layer of information for filtering, the fundamental question to be answered, especially in the context of gene discovery, is if a specific variant or mutated gene is indeed the disease-causing one [135,136]. To address this complex question, a new generation of tools is being developed, that instead of merely excluding information, perform variants ranking thereby allowing their prioritization. Different approaches have been proposed. For instance, PHIVE explores the similarity between human disease phenotype and those derived from knockout experiments in animal model organisms [137]. Other algorithms attempt to solve this problem by an entirely different way, through the computation of a deleteriousness score (also known as burden score) for each gene, based on how intolerant genes are to normal variation and using data from population variation databases [138]. It was proposed that human disease genes are much more intolerant to variants than non-disease associated genes [139,140]. The human phenotype ontology (HPO) enables the hierarchical sorting by disease names and clinical features (symptoms) for describing medical conditions. Based on these descriptions, HPO can also provide an association between symptoms and known disease genes. Several tools attempt to use these phenotype descriptions to generate a ranking of potential candidates in variant prioritization. As an example, some attempt to simplify analysis in a clinical context, such as the phenotypic interpretation of exomes [141] that only reports genes previously associated with genetic diseases. While others can also be used to identify novel genes, such as Phevor [142] that use data gathered in other related ontologies, gene ontology (GO) for example, to suggest novel gene–disease associations. The main goal of these tools is to end-up with few variants to further validation with molecular techniques [143,144]. Recently, several commercial software has been developed to aid in interpretation and prioritization of variant in a diagnostic context, that is simple to use, intuitive and can be used by clinicians, geneticist and researchers, such as VarSeq/VSClinical (Golden Helix), Ingenuity Variant Analysis (Qiagen), Alamut^®^ software (interactive biosoftware) and VarElect [145]. Besides those tools that aid in interpretation and variant analysis, currently clinicians have at their disposal several medical genetics companies, such as Invitae (https://www.invitae.com/en/) and CENTOGENE (https://www.centogene.com/) that provide to clinicians a precise medical diagnosis.

## 5. NGS Pitfalls

Seventeen years have passed since the introduction of the first commercially available NGS platform, 454 GS FLX from Life Sciences. Since then the “genomics” field has greatly expanded our knowledge about structural and functional genomics and the underlying genetics of many diseases. Besides, it allows the creation of the concepts of “omics” (transcriptomic, genomics, metabolomic, etc.), which provide new insights into the knowledge of all living beings, to know how different organisms use genetics and molecular biology to survive and reproduce in healthy and disease situations, to know about their population networks and changes in environmental conditions. This information is very useful also to understand human health. It is clear that NGS brought a panoply of benefits and solutions for medicine and to other areas, such as agriculture that helped to increase the quality and productivity [34,150,151,152]. However, it has also brought new challenges.

The first challenge is regarding the sequencing costs. Although is true that the overall costs of NGS comparing with the gene-by-gene sequence of Sanger sequencing, an NGS experiment is not cheap and still not accessible to all laboratories. It imposes high initial costs with the acquisition of the sequencing machine, which can go from thousands to a hundred thousand euros depending on the type of machine, plus consumables and reagents. Costs with experimental design, sample collection and sequencing library preparation also must be taken into account. Moreover, many times costs with the development of sequencing pipelines, and the development of bioinformatical tools to improve those pipelines and to perform the downstream sequence analysis, as well as the costs of data management, informatics equipment and downstream data analysis, are not considered in the overall NGS costs. A typical BAM file from a single WES experience consumes up to 30 Gb of space, thus storing and analyze data of several patients requires higher computational power and, also storing space, which has clearly significant costs. Further, expert bioinformaticians may also be needed to deal with data analysis, especially when working with WGS projects. These additional costs are evidently part of NGS workflow and must be accounted for (for further reading on that topic we recommend [153,154]).

Concerns about data sharing and confidentiality may also arise with the massive amount of data that is generated with NGS analysis. Is debatable which degree of protection should be adopted to genomic data, should genomic data be or not be shared between multiple parties (including laboratory staff, bioinformaticians, researchers, clinicians, patients and their family members)? It is a very important issue especially in the clinician context [155].

**Figure 7:**
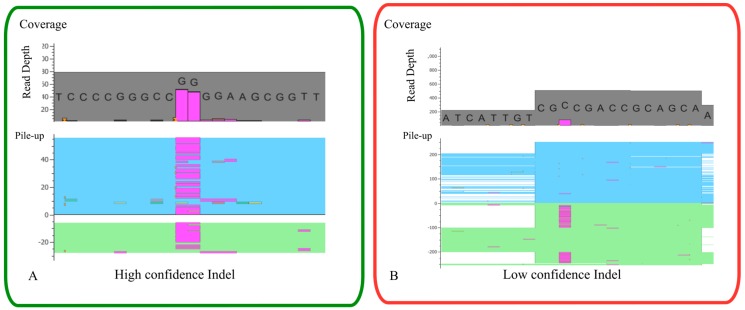
BAM (binary alignment map) file visual inspection. Two examples of situations that may be observed through this inspection. (**A**) Demonstrates a case of a true-positive INDEL, confirmed by Sanger sequencing. In contrast, (**B**) shows a clear example of a false-positive result, where the variant is present in only reverse reads, as later demonstrated by Sanger sequencing it is a technical artifact and should be excluded from further analysis.

When analyzing NGS data is important to be aware of its technical limitations, as highlighted throughout this paper, namely PCR amplification bias (a significant source of bias due to random errors that can be introduced), and sequencing errors and thus high coverage is needed to understand which variants are true and which are caused by sequencing or PCR errors. Further, limitations also exist in downstream analysis as an example of the read alignment/mapping, especially for indels in which some alignment tools have low detection capabilities or not detect at all [156]. Besides the bioinformatic tools that have helped and made the data analysis more automatic, a manual inspection of variants in the BAM file are frequently needed (Figure 7). Thus, is critical to understand the limitations of in-house NGS platform and workflow to try to overcome those limitations and increase the quality of variant detection.

One additional major challenge to clinicians and researchers is to correlate the findings with the relevant medical information, which may not be a simpler task, especially when dealing with new variants or new genes not previously associated with disease, which requires additional efforts to validate the pathogenicity of variants (which in a clinical setting may not be feasible). More importantly, both clinicians and patients must be clearly conscious that a positive result, although providing an answer that often terminates a long and expensive diagnostic journey, does not necessarily mean that a better treatment will be offered nor that it will be possible to find a cure, hence, still in many cases that genetic information will not alter the prognosis or the outcome for an affected individual [157]. This is an inconvenient and hard true that clinicians should clearly explain to patients. Nevertheless, huge efforts have been made to increase the choice of the best therapeutic options based on DNA sequencing results, namely for cancer [158] and a growing number of rare diseases [144,159].

## 6. Conclusions

To conclude, despite all the accomplishments made so far, a long journey is ahead before genetics can provide a definitive answer towards the diagnoses of all genetic diseases. Further improvements in sequencing platforms and data handling strategies are required, in order to reduce error rates and to increase variant detection quality. It is now widely accepted that in order to increase our understanding about the disease, especially the complex and heterogeneous diseases, scientists and clinicians will have to combine information from multiple -omics sources (such genome, transcriptome, proteome and epigenome). Thus, the NGS is evolving rapidly to not only deal with the classic genomic approach but are rapidly gaining broad applicability [160,161]. However, one major challenge is to deal with and interpret all the distinct layers of information. The current computational methods may not be able to handle and extract the full potential of large genomic and epigenomic data sets being generated. Above all, (bio)informaticians, scientists and clinicians will have to work together to interpret the data and to develop novel tools for integrated systems level analysis. We believe that machine learning algorithms, namely neural networks and support vector machines, as well as the emerging developments in artificial intelligence, will be decisive to improve NGS platforms and software, which will help scientists and clinicians to solve complex biological challenges, thus improving clinical diagnostics and opening new avenues for novel therapies development.

## Figures and Tables

**Table 1:** List with examples of widely used tools to perform an NGS functional filter.

Software	Short Description	Ref.
PhyloP Phylogenetic *p*-values	Based on a model of neutral evolution, the patterns of conservation (positive scores)/acceleration (negative scores) are analyzed for various annotation classes and clades of interest.	[146]
SIFT Sorting Intolerant from Tolerant	Predicts based on sequence homology, if an AA substitution will affect protein function and potentially alter the phenotype. Scores less than 0.05 indicating a variant as deleterious.	[112]
PolyPhen-2 Polymorphism Phenotyping v2	Predicts the functional impact of an AA replacement from its individual features using a naive Bayes classifier. Includes two tools HumDiv (designed to be applied in complex phenotypes) and HumVar (designed to diagnostic of Mendelian diseases). Higher scores (>0.85) predicts, more confidently, damaging variants.	[113]
CADD Combined Annotation Dependent Depletion	Integrates diverse genome annotations and scores all human SNV and Indel. It prioritizes functional, deleterious, and disease causal variants according to functional categories, effect sizes and genetic architectures. Scores above 10 should be applied as a cut-off for identifying pathogenic variants.	[114]
MutationTaster	Analyses evolutionary conservation, splice-site changes, loss of protein features and changes that might affect the amount of mRNA. Variants are classified, as polymorphism or disease-causing	[147]
Human Splice Finder	Predict the effects of mutations on splicing signals or to identify splicing motifs in any human sequence.	[133]
nsSNPAnalyzer	Extracts structural and evolutionary information from a query nsSNP and uses a machine learning method (Random Forest) to predict its phenotypic effect. Classifies the variant as neutral and disease.	[148]
TopoSNP Topographic mapping of SNP	Analyze SNP based on its geometric location and conservation information, produces an interactive visualization of disease and non-disease associated with each SNP.	[149]
Condel Consensus Deleteriousness	Condel integrates the output of different methods to predict the impact of nsSNP on protein function. The algorithm based on the weighted average of the normalized scores classifies the variants as neutral or deleterious.	[115]
ANNOVAR * Annotate Variation	Annotates the variants based on several parameters, such as identification whether SNPs or CNVs affect the protein (gene-based), identification of variants in specific genomic regions outside protein-coding regions (region-based) and identification of known variants documented in public and licensed database (filter-based)	[116]
VEP * Variant Effect Predictor	Determines the effect of multiple variants (SNPs, insertions, deletions, CNVs or structural variants) on genes, transcripts and protein sequence, as well as regulatory regions.	[117]
snpEff *	Annotation and classification of SNV based on their effects on annotated genes, such as synonymous/nsSNP, start or stop codon gains or losses, their genomic locations, among others. Considered as a structural based tool for annotation.	[118]
SeattleSeq *	Provides annotation of SNVs and small indels, by providing to each the dbSNP rs IDs, gene names and accession numbers, variation functions, protein positions and AA changes, conservation scores, HapMap frequencies, PolyPhen predictions and clinical association.	[119]

AA—amino acid; SNV—single nucleotide variant, Indel—small insertion/deletion variants, SNP—single nucleotide polymorphism, nsSNP—nonsynonymous SNP; CNV—copy number variation; * these tools, although also able to filter variants, are primarily responsible for variant annotation.

## Supplementary Materials

The following are available online at https://www.mdpi.com/2077-0383/9/1/132/s1, Figure S1: Summary of the tree main genome assemblers’ methods and the most widely used software that applies each method.
