# Supplementary material for: Bioinformatics and Computational Tools for Next-Generation Sequencing Analysis in Clinical Genetics
Source: J Clin Med. 2020 Jan 3;9(1):132. doi: 10.3390/jcm9010132 (PMC7019349; doi:10.3390/jcm9010132)
Supplement: Supplementary file 1 [file jcm-09-00132-s001.pdf]

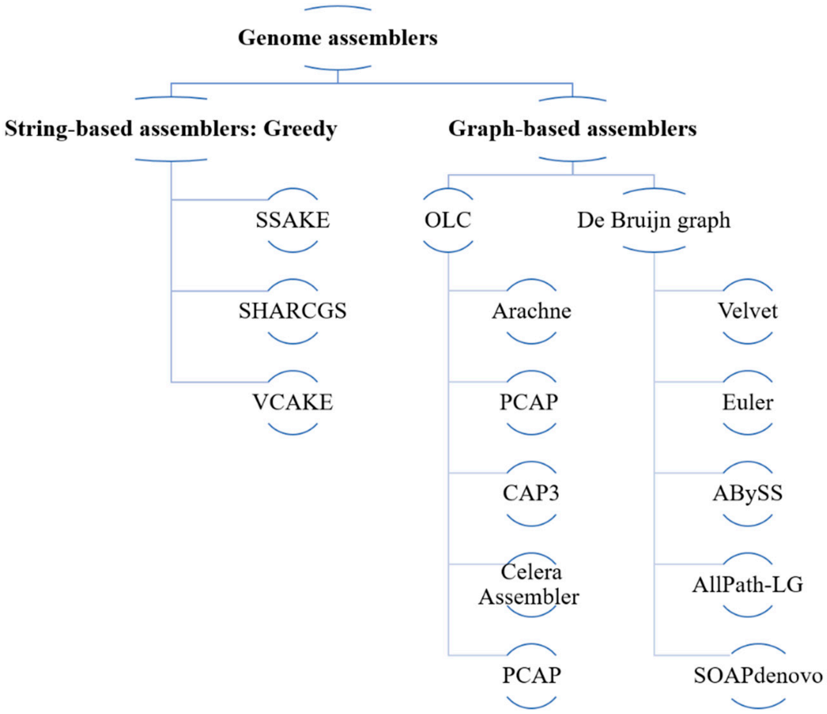

Figure S1. Summary of the tree main genome assemblers' methods and the most widely used software that applies each method.
